# Cross-cultural validation of the SNOT-22 questionnaire in the Colombian population

**DOI:** 10.1016/j.bjorl.2026.101801

**Published:** 2026-03-19

**Authors:** Susana Soto-Tirado, Silvia Nicté Villatoro-Rodríguez, Tania Margarita Salgado, Lina Paola Londoño-Martínez, Benjamín Darío Ariza de la Ossa, Juan David Bedoya-Gutiérrez

**Affiliations:** a University of Antioquia, Antioquia, Colombia; b Alma Mater Hospital of Antioquia, Antioquia, Colombia; c Universidad del Valle, Valle del Cauca, Colombia

**Keywords:** Rhinosinusitis, Validation study, Signs and symptoms, Respiratory, Patient health questionnaire

## Abstract

**Objective:**

To validate and culturally adapt the SNOT-22 for use in patients with chronic rhinosinusitis in Colombia.

**Methods:**

A cross-sectional study was conducted to assess the content validity, construct validity, and reliability of the SNOT-22 in a Colombian sample. Cultural adaptation involved expert review and patient testing to ensure comprehension and relevance.

**Results:**

Item adaptation and comprehension were achieved through consensus by six expert rhinologists and subsequent validation with 15 otorhinolaryngologists and 24 patients. The third item was modified for better comprehension. Content validity indices confirmed the questionnaire’s relevance, sufficiency, and comprehension, though some redundancy was noted in emotional and ear/facial symptom domains. In a sample of 221 completed questionnaires, polychoric correlations showed positive inter-item relationships. Internal consistency was excellent (Cronbach’s α = 0.97; ω = 0.96), and the model demonstrated good fit. Convergent validity with the Visual Analog Scale showed a Spearman correlation of 0.74. The questionnaire also showed strong discriminative validity, differentiating patients with and without rhinosinusitis (mean score difference of 49-points; Mann-Whitney effect size = 0.41). The Area Under the Curve (AUC) was 0.92, with 71.5% sensitivity and 4.8% false positive rate.

**Conclusion:**

The Colombian version of the SNOT-22 demonstrated strong patient comprehension, content validity, high internal consistency, and effective discriminative capacity. These results support its use as a reliable clinical tool for evaluating chronic rhinosinusitis in the Colombian population.

**Level of evidence:**

III.

## Highlights

• Culturally adapted the SNOT-22 for Colombian patients with expert input.
• High internal consistency: Cronbach’s α = 0.97, omega = 0.96.
• Strong correlation with symptom severity (VAS, Spearman *r* = 0.74).
• Effectively discriminate patients with and without rhinosinusitis.
• High diagnostic accuracy: AUC = 0.92, sensitivity = 71.5%.

## Introduction

Rhinosinusitis (RS) is the inflammation of the nasal mucosa and paranasal sinuses that significantly impacts the patient’s health and quality of life. It has been compared to other serious diseases such as angina pectoris, heart failure and chronic obstructive pulmonary disease.^1^ In the United States, RS symptoms account for 2% of medical consultations.^2^^,^^3^ In Colombia, between 2009 and 2014, 73.62% of consultations, emergency and hospitalization services were due to respiratory diseases, with chronic sinusitis occupying third place of these consultations.^4^^,^^5^

To diagnose Chronic Rhinosinusitis (CRS) in adults, the criteria of the European Position Paper on Rhinosinusitis and Nasal Polyps (EPOS) 2020 and 20232,^3^ must be met. These guidelines, the Clinical Practice Guidelines for the treatment of CRS of the Colombian Association of Otolaryngology of 2023^6^ and the International Consensus on Allergy and Rhinology 2021,^7^ recommend the use of the Sino-nasal Outcome Test-22 (SNOT-22) and the Visual Analog Scale (VAS) of nasosinusal symptoms as tools to objectify symptoms, taking into account that the VAS includes symptoms of asthma and allergic rhinitis; while SNOT has more specific symptoms of CRS.^7^, ^8^, ^9^, ^10^

The SNOT-22, developed and validated in England,^11^^,^^12^ is freely available and its use is not restricted.^11^^,^^12^ It has been validated in multiple languages and countries, including Spanish-speaking countries such as Spain and Chile.^13^, ^14^, ^15^ The questionnaire consists of 22-items, divided into 5-domains: nasal, ear/facial, sleep, functionality and emotions. It has been used at the time of diagnosis, clinical evolution, postoperative follow-up and as a predictor of improvement in patients undergoing surgery for CRS.^9^^,^^16^

Although the SNOT-22 is recommended for its use in Colombia,^5^^,^^6^ the cultural, linguistic and demographic differences between the Colombian population^17^ and those in which the questionnaire has been previously validated and ‒ coupled with Spanish's regional dialects, as noted in David E. Eddington's 2022 study from the Autonomous University of Barcelona^18^ ‒ justify a validation process. This study seeks to validate the content, construct and reliability of the SNOT-22 questionnaire by experts and by patients with CRS in the Colombian population.^13^

## Methods

Cross-sectional validation study of the SNOT-22 instrument.

It was performed at XXXXX, at the XXXXX, XXXXX and ENT consultations (Supplementary Material) in Hospital Alma Máter de Antioquia from June to October 2024.

Inclusion criteria: Patients over 18-years of age who met EPOS criteria for a diagnosis of CRS.

Exclusion criteria: Patients with a history of nasosinusal cancer.

### Instrument

SNOT-22, the version validated in Chile was used as a starting point.^13^ Each item was scored from 0 to 5 by the patient, being 0 no problem, 1 very slight problem, 2 slight problem, 3 moderate problem, 4 severe problem and 5 problem as bad as it can be. It can receive a minimum score of 0 and a maximum of 110. Considering that more than 40-points show a severe affectation,^2^^,^^3^ moderate between 20- and 40-points, and mild between 8- and 20-points.^2^^,^^3^^,^^19^

VAS for nasosinusal symptom severity, a psychometric instrument that graphically evaluates the patients' symptoms, with a 10 cm line, where the patients drew a vertical line from 0 to 10 according to the discomfort generated by the symptoms, where 0 represents none and 10, more than they can imagine (Supplementary Material).^20^

### Sample size

A different sample was required for each stage of the study. The comprehension and content validity assessment were carried out with 5 rhinology experts and 10 Colombian otorhinolaryngologists and a comprehension assessment by patients during the pilot test.

Subsequently, for construct validity, a minimum of 10 patients per item was calculated for the confirmatory factor analysis, which in this case corresponds to 220 patients, which was considered sufficient for scale validation studies.^21^, ^22^, ^23^, ^24^, ^25^, ^26^, ^27^ A loss rate of 20% was estimated, which was intended to recruit 264 patients.

For the McDonald omega coefficient, which uses item loadings, the sample size calculation of the confirmatory factor analysis was considered sufficient.^28^

### Sampling criteria

For construct validity, convenience sampling was performed. Patients with a diagnosis of CRS were included as they arrived for consultation with specialists from June 2024.

For further patient collection, the databases of otolaryngology and allergology consultations in XXXXX and rhinology in Hospital Alma Máter de Antioquia from June to October 2024 were used.

### Cross-cultural adaptation and content validity assessment

The study consists of two important components, for the first one, which is the cross-cultural adaptation, comprehension and content validity, the questionnaire is applied to patients diagnosed with CRS and to experts, and the comprehension of the questions is evaluated through a survey, being 10-totally easy to understand and 0-incomprehensible (Supplementary Material), it is recorded if the patients required explanation of any item or statement and if there was any question that was not understood and how it would be improved.

For each question, a score above 7 means that the item is accepted. A score of 4 to 6 causes the item to be discussed by experts. If a score of 1 to 3, the item is changed by expert consensus through the Delphi method (Supplementary Material).

An evaluation of the content validity was carried out with the experts, for which each of the items and domains is qualified in its sufficiency, relevance and completeness, assigning a score from 1 to 3, being 3 that the item is essential to evaluate the construct; 2, that it results or 1, that it is considered unnecessary.

### Construct validity and internal consistency

Sociodemographic and clinical data were collected by experts (rhinologists), allergists and otorhinolaryngologists collaborating during the study period in XXXXX, XXXXX, XXXXX and private offices (Supplementary Material). Patients self-completed the VAS, SNOT-22, and questions were answered if necessary.

For further collection of patients, the databases of all otolaryngology and allergology consultations at XXXXX and rhinology at XXXXX from April to October 2024 were used, where each medical record was searched, filtering patients who met the criteria and interviewing them by telephone for SNOT-22 and sociodemographic data; clinical data were taken from clinical records.

### Statistical analysis

For content validity, the modified Content Validity Ratio (CVR) and Content Validity Index (CVI)^29^ were calculated with these data. Scores above 0.58 are required to be considered as exceeding the validation threshold in a 15-judge study.^27^^,^^28^

For construct validity, the proposed model with 6 domains and 22 items was used. The factor loadings, the correlation between factors were estimated and the fit of the model to the data was evaluated with goodness-of-fit indices: chi2 test, CFI (Comparative fit index, *≥*0.95: Good fit. Between 0.90 and 0.95: Acceptable. < 0.90: Poor fit), SRMR (Standardized root mean square residual < 0.08: Adequate fit. SRMR < 0.05: excellent fit), TLI (Tucker-Lewis index *≥* 0.95: good fit. Between 0.90 and 0.95: acceptable fit. < 0.90: poor fit), RMSE (Root mean squared error < 0.05: excellent fit. < 0.08: Reasonable fit. < 0.10: Questionable fit. ≥ 0.10: Poor fit).

For the evaluation of internal consistency, the alpha, G6 and omega coefficients were evaluated in each of the dimensions and in the complete questionnaire.^26^^,^^27^ Between 0.70 and 0.90 were indicative of adequate internal consistency.

Convergence validity was performed by correlating the scores of the SNOT-22 questionnaire with those obtained by the VAS of nasosinusal symptoms.^29^ Moderate to strong correlations were expected. In addition, the discriminative validity of the tool was evaluated by comparing the values obtained in patients with CRS and without CRS. The Wilcoxon–Mann–Whitney tests were performed, and the effect size was estimated with the Mann Whitney statistics. Associations were expected to be weak. For this purpose, a Receiver Operating Characteristic (ROC) curve was elaborated, in which it was expected to find an area under the curve greater than or equal to 0.70 to consider that the questionnaire was able to adequately discriminate between sick and healthy patients.^31^

The Data were collected in Excel databases and for descriptive analyses, confirmatory factor analysis, goodness of fit, alpha, omega, and G6 values, convergent and discriminative validity, the *R* statistic was used.

### Software

R software version 4.4.1 (2024-06-14 ucrt) and RStudio version 2024.09.1 + 394 were used for this statistical analysis and graphics. Native packages such as stats, graphics and others from collaborators such as DescTools and summarytools, psych, MBESS, ufs and lavaan, mltools, Metrics, ROCR, dplyr, ggplot2 and tidyverse were used.^29^, ^30^, ^31^, ^32^, ^33^, ^34^, ^35^, ^36^, ^37^

## Results

### Comprehension

In the evaluation of comprehension by the experts, it was found that item 3: *Continuous nasal mucus*, of the SNOT-22 available in Spanish,^13^ was not comprehensible (Table 1). In unanimous consensus of 6 rhinologists, it was decided that the most comprehensible way to communicate the item would be *Continuous nasal discharge* (*Secreción nasal continua*), and this adaptation was made to the questionnaire (Supplementary Material).

**Table 1:** Experts SNOT-22 comprehension.

Domino	Item	Statement Spanish	Statement English	Mean in Pilot Test	Mean in adapted SNOT-22
Header				7.4	8.2
Nose	Item 1	Necesidad de sonarse la nariz	Need to blow nose	10.0	10.0
	Item 2	Estornudos	Sneezing	10.0	10.0
	Item 3	Secreción nasal continua (en piloto, mucosidad nasal continua)	Runny nose	6.4	9.5
	Item 4	Tos	Cough	9.6	9.9
	Item 5	Cae secreción por atrás hacia la garganta	Post-nasal discharge	8.2	8.8
	Item 6	Secreción nasal espesa	Thick nasal discharge	9.4	9.5
	Item 21	Obstrucción nasal	Nasal Blockage	10.0	9.9
Ears/Facial	Item 7	Sensación de oído tapado	Ear fullness	10.0	10.0
	Item 8	Mareos	Dizziness	9.6	9.3
	Item 9	Dolor de oído	Ear pain	10.0	10.0
	Item 10	Presión o dolor en la cara	Facial pain/pressure	9.8	9.9
Sleep	Item 11	Dificultad para quedarse dormido (a)	Difficulty falling asleep	9.2	9.7
	Item 12	Se despierta durante la noche	Wake up at night	8.8	9.5
	Item 13	Sensación de que durmió mal	Lack of a good night’s sleep	8.2	9.0
	Item 14	Despertar cansado (a)	Wake up tired	8.2	9.2
Functionality	Item 15	Fatiga o cansancio	Fatigue	9.0	9.4
	Item 16	Productividad o rendimiento disminuido	Reduced productivity	8.6	9.1
	Item 17	Menor o poca concentración	Reduced concentration	9.0	9.4
Emotions	Item 18	Frustración, cansancio, irritabilidad	Frustrated/restless/irritable	9.2	9.5
	Item 19	Triste	Sad	7.8	9.3
	Item 20	Sentirse avergonzado	Embarrassed	8.2	9.3
Sense of smeel and taste	Item 22	Pérdida del sentido del olfato y gusto	Decreased Sense of Smell/Taste	9.4	9.8

The adapted SNOT-22 was administered to 15 otolaryngologists, who found it *easy to understand*, with all items having values above 8 (Supplementary Material).

The adapted SNOT-22 was applied to 24 patients with CRS and an average comprehension value of 9.62 was obtained for both the statement and the items, with only one patient requiring explanation or clarification of the statement (Supplementary Material).

### Content validity

With the evaluation of 15 experts/judges, a modified CVR greater than 0.58 was obtained, reaching the relevance threshold in 14 of the items (Table 2).

**Table 2:** Content Validity Ratio (CVR).

Domain	Item	Relevance CVR	Exhaustivity CVR	Sufficiency CVR
Nose	Item 1	0.86	0.93	0.93
	Item 2	0.73		
	Item 3	1.00		
	Item 4	0.53		
	Item 5	1.00		
	Item 6	0.73		
	Item 21	1.00		
Ears/Facial	Item 7	0.53	0.60	0.66
	Item 8	0.26		
	Item 9	0.26		
	Item 10	0.93		
Sleep	Item 11	0.66	0.66	0.66
	Item 12	0.60		
	Item 13	0.66		
	Item 14	0.60		
Functionality	Item 15	0.60	0.73	0.60
	Item 16	0.60		
	Item 17	0.46		
Emotions	Item 18	0.46	0.53	0.53
	Item 19	0.40		
	Item 20	0.53		
Sense of smell and taste	Item 22	1.00	0.73	0.73

The domains of nose, ears/facial, sleep, functionality, and sense of smell and taste had CVR greater than 0.58, exceeding the threshold for completeness and sufficiency in patients with CRS.

The content CVIs for the questionnaire were 0.65 for relevance, 0.74 for sufficiency and 0.69 for completeness, being above the 0.58 threshold.

### Validity and reliability

#### Characteristics of the participants

A total of 216 patients in Medellin, 8 patients in Cali and 11 patients in Bogota were collected between June and October 2024. A total of 235 applications of the SNOT-22 were made, 14 of which were excluded because they did not meet the diagnostic criteria or because fundamental data for such diagnosis was missing. Finally, there were 221 applications of the questionnaire for analysis, with the sociodemographic and clinical characteristics described (Table 3).

**Table 3:** Population characteristics.

Category		n°	%
Age (years)	18‒27	20	9.0
	28‒37	38	17.2
	38‒47	61	27.6
	48‒57	40	18.1
	58‒67	41	18.6
	68‒77	18	8.1
	78‒87	3	1.4
Medical History	Asthma	64	28.9
	None	59	26.7
	Hypothyroidism	29	13.1
	Hypertension	29	13.1
	Dyslipidemia	18	8.1
	Allergic Rhinitis	12	5.4
	Sleep Apnea	11	4.9
	AERD	9	4.1
	Migraine	9	4.1
	Diabetes	7	3.2
	Depression	6	2.7
	COPD	5	2.3
	Fibromyalgia	5	2.3
	Cystic Fibrosis	2	0.9
Previous Surgery		117	53.2
Nasal Obstruction		196	89.5
Nasal Congestion		201	91.0
Postnasal Drip		190	86.0
Facial Pain		162	73.3
Anosmia		175	79.9
RS Findings on Nasolaryngoscopy		167	75.1
RS Findings on CT Scan		182	82.4
Survey Format	In-person	80	36.2
	Phone	141	63.8
Medications	Nasal Steroid	150	67.8
	Antihistamine	91	41.2
	Dupilumab	22	9.9
	Montelukast	35	15.8
	Pulmonary Inhalers	60	27.1
	Immunotherapy	3	1.3
	Omalizumab	1	0.4
	Mepolizumab	1	1.3

AERD, Aspirin-Exacerbated Respiratory Disease; COPD, Chronic Obstructive Pulmonary Disease; RS, Rhinosinusitis.

#### Construct validity (structural)

**Fig. 1:**
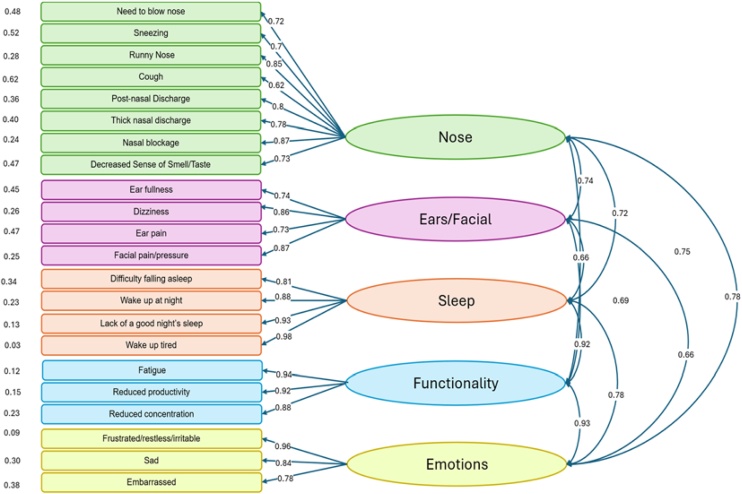
Confirmatory Factor Analysis. Factor loadings between domains and items are shown. Errors to the left of the items are shown.

From the results of the 221 SNOT-22 applied, 6 item values had to be imputed, which were calculated by averaging what was obtained in the domain of the missing data. These values were used to calculate the polychoric correlation matrix (Supplementary Material), the reliability analysis (Table 4), the confirmatory factor analysis (Fig. 1) and the goodness of fit indices.

**Table 4:** Reliability analysis by domains.

Domain	Alpha	CI alpha	G6	Omega	CI Omega
Nose	0.89	0.87‒0.91	0.90	0.92	0.87‒0.14
Ears / Facial	0.83	0.79‒0‒87	0.79	0.84	0.79‒0.86
Sleep	0.92	0.89‒0.94	0.91	0.94	0.90‒0.94
Functionality	0.91	0.88‒0.93	0.88	0.91	0.88‒0.94
Emotions	0.86	0.82‒0.89	0.81	0.86	0.81‒0.89
SNOT-22 Adapted	0.95	0.94‒0.96	0.97	0.96	0.95‒0.96

The Polychoric Correlation plot (Supplementary Material) showed a positive correlation between all items (greater than 0), with higher correlation between items in the same domain.

The fit indices were as follows: CFI = 0.98, TLI = 0.97, SRMR = 0.49, RMSEA = 0.08 (95% CI: 0.071–0.090), Chi-Square = 483, p-value = 0, with 199 degrees of freedom.

### Comparison groups

#### Convergent validity

**Fig. 2:**
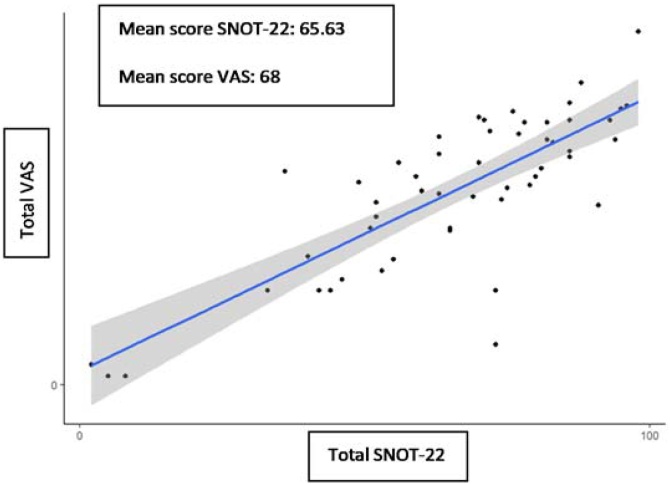
Scatter plot between SNOT-22 and VAS of nasosinusal symptoms.

53 VAS applications of nasosinusal symptoms were performed on patients with CRS and a Spearman correlation coefficient of 0.74 and positive linear correlation was determined with the scores acquired by those patients on the SNOT-22 (Fig. 2).

#### Discriminative validity

Assessed by administering the SNOT-22 to 26 healthy individuals and comparing their scores to those of patients with chronic rhinosinusitis. The median score of patients without CRS was 12 (IQR = 13) and the median score of patients with CRS was 61 (IQR = 46), with a difference of 49-points in the medians.

The Wilcoxon-Mann Whitney nonparametric test was performed, obtaining a value of W = 338 and a p-value of 5.036e-11. The effect size was estimated with the Mann Whitney statistic which was 0.41 (moderate).

**Fig. 3:**
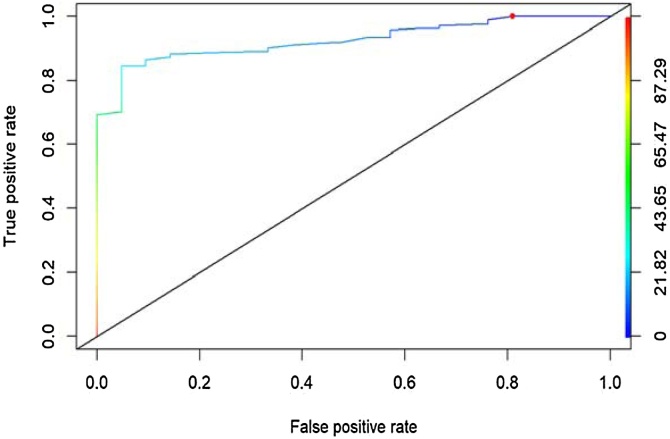
Area under the curve.

The area under the curve of the false positive and true positive rate between patients with and without CRS was 0.92. With a cutoff point of 40 points in the total score of the questionnaire, the false positive rate is 4.8% and the sensitivity is 71.5% (Fig. 3).

## Discussion

The Spanish version of the SNOT-22 questionnaire adapted in Colombia was found to be understandable by patients and experts, as well as reliable and valid. It required the adaptation of the item 3, from *Continuous nasal mucus* to *Continuous nasal discharge (Mucosidad nasal continua* a *Secreción nasal continua*) by expert criteria, with a unanimous decision to change. This form of the item coincides with the SNOT-22 validated in the Spanish population.^14^

Evaluating the content validity, it was found that 14 of the items were considered essential by the authors. The items coughing, feeling stuffy ear, dizziness, ear pain, less or poor concentration, frustration, tiredness, irritability, sadness, feeling embarrassed, were considered essential for more than half of the experts.^28^ The domains of nose, ears/facial, sleep, functionality, and sense of smell and taste were sufficient and comprehensive for assessing the symptoms of patients with CRS. Nevertheless, the completeness and sufficiency of the emotion domain was considered essential for more than half of the experts, and useful but not essential for some of the experts.

In the CVI of the complete SNOT-22 questionnaire, the content was found to be relevant, sufficient, and comprehensive. Additionally, in the reliability analysis, we see alpha and omega indices above 0.9, which indicates high internal consistency, but also suggests some redundancy of some of the items, mainly in the domains of ears/facial and emotions. Regardless of this possible redundancy, we decided to keep the 22 original items since more than half of the experts punctuated these items as essential. The questionnaire shows adequate content validity index because they have a high G6 index, which reflects that the items are related to the factor or construct, and its ease to facilitate their international applicability. Cronbach's alpha values were like those found in the validation in Chile and Spain, where values higher than 0.95 and 0.91, respectively, were found.^13^^,^^14^

Regarding the domain structure, a good fit was found, In the SNOT-22 study validated in Chile, multivariate normality was not found and therefore the factor analysis and goodness-of-fit adjustments were not pursued.^13^

As for the convergent validity we can see that the adapted SNOT-22 questionnaire and VAS of nasosinusal symptoms, which indicates that both tools measure similar constructs. Generally, a high SNOT-22 score will correspond to a high VAS score. In the validations available in Spanish, this comparison is not included, and we do not have references in this aspect.^13^^,^^14^

In the convergent validity, 3 atypical patients were found, 2 of them with higher comparative values of SNOT-22 with respect to VAS. One patient with insomnia, another with hypothyroidism, both with impairment in the domains of functionality and sleep. In cases of non-compensated hypothyroidism, we can expect that these domains can be affected, and this is evidenced in a systematic review of 2024, which relates thyroid function and sleep patterns.^38^ This information may explain why these patients could present high values in the SNOT-22 questionnaire.

The third atypical patient presented an atypical allergic pathology, with pulmonary and ocular involvement, which explained the higher in the VAS. This scale evaluates symptoms such as itchy eyes, tearing, chest tightness and wheezing, while the SNOT-22, due to its focus in nasosinusal symptoms, does not evaluate these aspects.

In the discriminative validity in patients without CRS and with CRS, mean scores of 11.6 and 57.9 respectively were found, while in the Chilean validation, mean values of 19.6 and 43.1 points were found, with significant differences between the groups. The greater mean differences in our population could be explained by a greater severity of the pathologies or by health centers that treat patients with complex pathologies.

In the discriminative validity, an outlier was seen in a patient without CRS, who presented a score of 43 in the SNOT-22 questionnaire. On reviewing his clinical data, it is found that he suffers from inflammatory bowel disease and his high score is due to the domains of functionality and sleep. Considering that 48% of patients with inflammatory bowel disease reported having nasal symptoms,^39^ and that a study conducted in Germany reported that 63% of those with inflammatory bowel disease had fatigue,^40^ we understand why this patient could present these values.

In the area under the curve, we see that the questionnaire discriminates between healthy and unhealthy patients with a value of 0.92, which indicates an excellent discriminative capacity. This value is superior to the values reported in the Chilean validation (AUC = 0.86).^13^

Amongst the limitations of our study, we found that the test-retest reproducibility test was difficult to perform. This was due to the high variability of symptomatology in short periods with the treatments, which do not allow us to control recall biases or the stability of the construct. Running two measurements in the same consultation would imply a recall bias and doing it in successive days would not be valid due to the treatment initiation or modification.

**Fig. 4:**
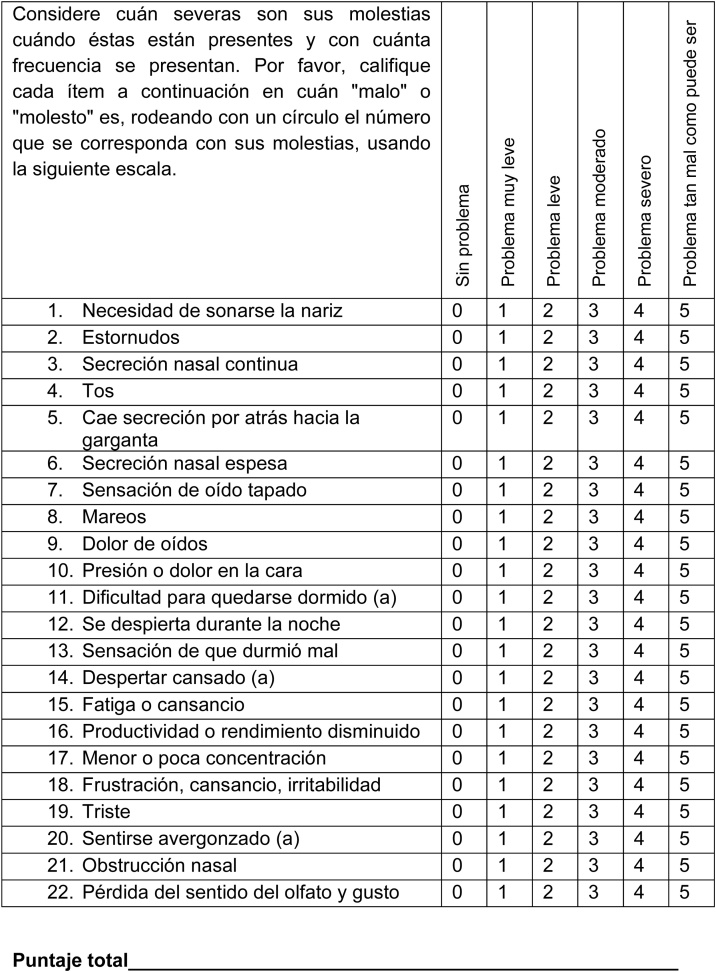
SNOT-22 Validated in the Colombian population in Spanish. CUESTIONARIO DE SÍNTOMAS NASOSINUSALES - SNOT 22 (SINO-NASAL-OUTCOME-TEST) VALIDADO EN POBLACIÓN COLOMBIANA Fecha: A continuación, usted encontrará una serie de síntomas y alteraciones socio/emocionales asociadas a su rinosinusitis. Nos gustaría saber más acerca de estos problemas y le solicitamos contestar las siguientes preguntas, lo mejor posible. No hay respuestas correctas o incorrectas y sólo usted nos puede entregar esa información. Por favor, califique sus molestias según cómo han sido estas últimas dos semanas. Gracias por su participación. No dude en pedir ayuda sí la necesita.

One of the strengths of this study is the inclusion of patients from different regions of Colombia, providing broader geographical and cultural representation of the population and supporting the external validity and generalizability of the findings to regions with similar sociodemographic characteristics. Although most participants were recruited in a single city, efforts were made to include patients from other regions, which adds value to the interpretation and applicability of the results (Fig. 4).

## Conclusions

The adapted Spanish version of the SNOT-22 questionnaire in the Colombian population is easy to understand by patients, was evaluated by experts as exhaustive, relevant and sufficient. It shows convergent and discriminative validity, with good reliability, which supports its utility as a valid instrument for clinical evaluation in patients with chronic rhinosinusitis in Colombia.

The domain of emotions is the one that presents the greatest doubt in its completeness, relevance and sufficiency, without affecting the content validity indexes of the content validation which were close to the accepted thresholds of CVR.

Patients with pathologies that affect functionality and sleep, such as insomnia, hypothyroidism or inflammatory bowel disease, were identified, and could have higher scores in the SNOT-22 questionnaire mainly in these domains, which must be considered during the clinical interpretation of the results.

## ORCID ID

Susana Soto-Tirado: 0000-0002-1533-7293

Silvia Nicté Villatoro-Rodríguez: 0009-0003-2608-8127

Tania Margarita Salgado: 0000-0002-9742-2570

Lina Paola Londoño-Martínez: 0009-0009-2851-8008

Benjamín Darío Ariza de la Ossa: 0000-0002-1083-7889

Juan David Bedoya-Gutiérrez: 0000-0002-1009-1781

## Funding

Self-funding and epidemiology professors funded by the University of Antioquia. Collaborating general practitioner in data collection, financed by Hospital Universitario Alma Mater.

## Data availability statement

The authors declare that all data are available in repository.

## Declaration of competing interest

The authors declare no conflicts of interest.
