# Supplementary material for: Cross-cultural validation of the SNOT-22 questionnaire in the Colombian population
Source: Braz J Otorhinolaryngol. 2026 Mar 19;92(3):101801. doi: 10.1016/j.bjorl.2026.101801 (PMC13019075; doi:10.1016/j.bjorl.2026.101801)
Supplement: Supplementary file 1 [file mmc1.docx]

BJORL-D-25-00314_Material Supplementary

**Estudio “Validación Transcultural Del Cuestionario SNOT-22 En Población Colombiana”**

**MATERIAL SUPLEMENTARIO 1. CONSENTIMIENTO Y GUIÓN**

**CONSENTIMIENTO INFORMADO**

Este es un estudio clínico considerado según la resolución 008430 (artículo 11) de 1993 del  Ministerio de Salud de Colombia, como investigación sin riesgo, por tanto puede prescindir del consentimiento informado (artículo 16) , pero dados los datos demográficos y datos clínicos sensibles, se dispone del presente consentimiento.

**CONSENTIMIENTO INFORMADO**

Estimado/a participante

Le agradecemos su interés en participar en el estudio de investigación titulado "Validación Transcultural de la Encuesta SNOT-22 en Población Colombiana". Antes de que decida participar, es importante que comprenda los detalles del estudio, sus objetivos, los procedimientos involucrados, los posibles beneficios y riesgos, así como sus derechos como participante. Le recomendamos que lea detenidamente este documento y que haga todas las preguntas que considere necesarias antes de tomar una decisión.

Investigador Responsable:

Susana Soto Tirado. Residente de Otorrinolaringología de la Universidad de Antioquia. susana.sotot@udea.edu.co

Propósito del estudio: El propósito de este estudio es validar transculturalmente la Encuesta SNOT-22 en la población colombiana. La Encuesta SNOT-22 es un cuestionario utilizado para evaluar la calidad de vida relacionada con los síntomas nasosinusales. Al participar en este estudio, nos ayudará a entender cómo se puede adaptar esta encuesta para ser utilizada de manera efectiva en la población colombiana.

Procedimientos involucrados: Si decide participar en este estudio, se le solicitará que complete la Encuesta SNOT-22, la cual consta de una serie de preguntas sobre sus síntomas nasosinusales y cómo afectan su calidad de vida. También se le pedirá que proporcione información demográfica básica. El tiempo estimado para completar la encuesta es de aproximadamente 10 minutos

Beneficios de participar: Al participar en este estudio, usted contribuirá a la validación de la Encuesta SNOT-22 en la población colombiana, lo que puede ayudar a mejorar la atención médica y la calidad de vida de las personas con síntomas nasosinusales en Colombia. Si bien no hay beneficios directos garantizados para usted como participante, su participación es fundamental para el éxito de esta investigación.

Riesgos y molestias: Los riesgos asociados con la participación en este estudio son mínimos. La principal molestia puede ser el tiempo dedicado a completar la encuesta y la posibilidad de experimentar incomodidad o frustración al responder preguntas sobre sus síntomas nasosinusales.

Confidencialidad y protección de datos: Toda la información recopilada durante el estudio se mantendrá estrictamente confidencial. Su nombre y cualquier dato personal identificable no se divulgarán en ningún informe o publicación relacionada con este estudio. Solo se utilizarán datos agregados y anonimizados para el análisis estadístico.

Participación voluntaria: Su participación en este estudio es completamente voluntaria. Tiene derecho a retirarse en cualquier momento sin consecuencias negativas o penalizaciones. La decisión de participar o no en este estudio no afectará de ninguna manera su atención médica ni su relación con el equipo de investigación.

Contacto e información adicional: Si tiene alguna pregunta o inquietud sobre el estudio o su participación, no dude en comunicarse con el investigador responsable cuyos detalles se proporcionan anteriormente. También puede buscar asesoramiento adicional antes de tomar una decisión.

Consentimiento: Al firmar a continuación, confirmo que he leído y comprendido la información proporcionada en este documento de Consentimiento Informado. Se me ha brindado la oportunidad de hacer preguntas y se me han dado respuestas satisfactorias. Acepto participar en el estudio "Validación Transcultural de la Encuesta SNOT-22 en Población Colombiana" de forma voluntaria y comprendo que puedo retirarme en cualquier momento sin consecuencias negativas.

**(Firma del participante)**

**(Fecha)**

**(Firma del Testigo N°1)**

**(Firma del Testigo N°2)**

**(Firma del Médico)**

Nota: Se debe proporcionar una copia de este documento al participante una vez firmado.

Agradecemos su consideración para participar en este estudio de investigación. Su contribución es esencial para avanzar en el campo de la medicina y mejorar la atención médica para las personas con síntomas nasosinusales en Colombia.

**GUIÓN DE LLAMADA Y CONSENTIMIENTO VERBAL.**

Buenas__________

Mi nombre es___________

Lo llamo de parte del Hospital Alma Mater de Antioquia/ORLANT, como parte de una investigación de otorrinolaringología de la Universidad de Antioquia.

Usted tuvo una cita reciente y quisiéramos hacerle una encuesta de sus síntomas como parte de una investigación que se está realizando en el país. El estudio es de la "Validación Transcultural de la Encuesta SNOT-22 en Población Colombiana". Esta escala es un cuestionario utilizado para evaluar la calidad de vida relacionada con los síntomas nasosinusales.

Si decide participar, le haríamos una serie de preguntas sobre sus síntomas nasosinusales y cómo afectan su calidad de vida, alguna información clínica y social.

Esto tomaría aproximadamente 5 minutos.

Antes de que decida participar, es importante que comprenda los detalles del estudio, sus objetivos, los procedimientos involucrados, los posibles beneficios y riesgos, así como sus derechos como participante y que haga todas las preguntas que considere necesarias antes de tomar una decisión.

La investigador Responsable es:

Susana Soto Tirado. Residente de Otorrinolaringología de la Universidad de Antioquia. susana.sotot@udea.edu.co

Al participar en este estudio, usted contribuirá a la validación de la Encuesta SNOT-22 en la población colombiana, lo que puede ayudar a mejorar la atención médica y la calidad de vida de las personas con síntomas nasosinusales en Colombia. Si bien no hay beneficios directos garantizados para usted como participante, su participación es fundamental para el éxito de esta investigación.

Los riesgos asociados con la participación en este estudio son mínimos. La principal molestia puede ser el tiempo dedicado a completar la encuesta y la posibilidad de experimentar incomodidad o frustración al responder preguntas sobre sus síntomas nasosinusales.

Toda la información recopilada durante el estudio se mantendrá estrictamente confidencial. Por obligación de confidencialidad y privacidad de los datos según la normatividad vigente.

 Su nombre y cualquier dato personal identificable no se divulgarán en ningún informe o publicación relacionada con este estudio. Solo se utilizarán datos agregados y anonimizados para el análisis estadístico.

La información y los datos tendrán manejo según la normatividad vigente de protección de datos personales, en la cual se tiene la obligación de confidencialidad y privacidad.

Su participación en este estudio es completamente voluntaria. Tiene derecho a retirarse en cualquier momento sin consecuencias negativas o penalizaciones. La decisión de participar o no en este estudio no afectará de ninguna manera su atención médica ni su relación con el equipo de investigación.

Si tiene alguna pregunta o inquietud sobre el estudio o su participación, no dude en comunicarse con el investigador responsable cuyos detalles se proporcionan anteriormente. También puede buscar asesoramiento adicional antes de tomar una decisión.

Confirmá que ha escuchado y comprendido la información proporcionada. Se le ha brindado la oportunidad de hacer preguntas y se le han dado respuestas satisfactorias. Acepta participar en el estudio "Validación Transcultural de la Encuesta SNOT-22 en Población Colombiana" de forma voluntaria y comprende que puedo retirarme en cualquier momento sin consecuencias negativas.

Comprende, esta de acuerdo y acepta participar

Si- No-

Muchas gracias por su participación.

**MATERIAL SUPLEMENTARIO. METODOLOGÍA**

**Expertos, rinologos Colombia:**

1. Dr. Juan David Bedoya Gutierrez. Universidad de Antioquia, Medellín juand.bedoya@udea.edu.co
2. Dr. Gustavo Adolfo Vanegas.  Universidad de Antioquia, Medellín gustavo.vanegas@udea.edu.co
3. Dra. Sara Vélez.  Universidad de Antioquia, Medellín saritopo@hotmail.com
4. Dr. Javier Ospina.  Fundación Santafe, Bogotá. ospinaotorrino@gmail.com
5. Dr. Ricardo Silva. Universidad Militar Nueva Granada, Bogotá. silvarueda@gmail.com
6. Dra. Carolina Mora.  Universidad Militar Nueva Granada, Bogotá  caromorad@gmail.com
7. Dr. Samuel Hernando Muñoz García. Cali.

**Otorrinolaringólogos y centros colaboradores para recolección de pacientes**

1. Hospital Alma Mater de Antioquia (En proceso de evaluación por parte del hospital)

- Servicio de Alergología
- Consulta externa de Otorrinolaringología

1. ORLANT

- Dr. Sebastián Orozco
- Dr. Juan David Bedoya Gutierrez.
- Dr. Gustavo Adolfo Vanegas
- Dr. Julián Molano
- Dra Sara Velez
- Dr Guillermo Arturo Landinez.
- Dra Xiomara Correa

1. Nasal IPS

- Dr. Juan David Bedoya Gutierrez.

1. Consultas externas

- Dr. Gustavo Adolfo Vanegas
- Dra. Carolina Mora
- Dr Andres Carvajal

**CUESTIONARIO DE SÍNTOMAS NASOSINUSALES - SNOT 22 (SINO-NASAL-OUTCOME-TEST). VALIDADO EN CHILE**

[https://www.scielo.cl/img/revistas/orl/v80n4//0718-4816-orl-80-04-0425-gf02.jpg](https://www.scielo.cl/img/revistas/orl/v80n4/0718-4816-orl-80-04-0425-gf02.jpg)

**PREGUNTAS DE COMPRENSIÓN DEL CUESTIONARIO PARA PACIENTES (12) EN PRUEBAS PILOTO**

Siendo 10 totalmente fácil de comprender y 0 incomprensible, califique de 0 a 10

¿Qué tan fácil fue la comprensión del enunciado de la escala?

________________________________________________________

¿Qué tan fácil fue la comprensión de las preguntas realizadas?

_________________________________________________________

¿Requirió que se le explicara o aclarara alguna pregunta de la escala?

SÍ 𛲡?   NO   𛲡 ¿Cual?______________________

**PREGUNTAS DE COMPRENSIÓN DE LA ESCALA PARA EXPERTOS (6) EN PRUEBAS PILOTO**

¿Qué tan fácil es la comprensión del enunciado de la escala?

0                                                                                                                               10

¿Qué tan fácil es la comprensión del ítem N° 1 "Necesidad de sonarse la nariz"?

0                                                                                                                               10

¿Qué tan fácil es la comprensión del ítem N° 2 "Estornudos"?

0                                                                                                                               10

¿Qué tan fácil es la comprensión del ítem N° 3 "Mucosidad nasal continua"?

0                                                                                                                               10

¿Qué tan fácil es la comprensión del ítem N° 4 "Tos"?

0                                                                                                                               10

¿Qué tan fácil es la comprensión del ítem N° 5 "Cae secreción por atrás hacia la garganta"?

0                                                                                                                               10

¿Qué tan fácil es la comprensión del ítem N° 6 "Secreción nasal espesa"?

0                                                                                                                               10

¿Qué tan fácil es la comprensión del ítem N° 7 "Sensación de oído tapado"?

0                                                                                                                               10

¿Qué tan fácil es la comprensión del ítem N° 8 "Mareos"?

0                                                                                                                               10

¿Qué tan fácil es la comprensión del ítem N° 9 "Dolor de oído”?

0                                                                                                                               10

¿Qué tan fácil es la comprensión del ítem N° 10 "Presión o dolor en la cara"?

0                                                                                                                               10

¿Qué tan fácil es la comprensión del ítem N° 11 "Dificultad para quedarse dormirdo (a)" ?

0                                                                                                                               10

¿Qué tan fácil es la comprensión del ítem N° 12 "Se despierta durante la noche"?

0                                                                                                                               10

¿Qué tan fácil es la comprensión del ítem N° 13 "Sensación de que durmió mal"?

0                                                                                                                               10

¿Qué tan fácil es la comprensión del ítem N° 14 "Despertar cansado (a)"?

0                                                                                                                               10

¿Qué tan fácil es la comprensión del ítem N° 15 "Fatiga o cansancio"?

0                                                                                                                               10

¿Qué tan fácil es la comprensión del ítem N° 16 "Productividad o rendimiento disminuido"?

0                                                                                                                               10

¿Qué tan fácil es la comprensión del ítem N° 17 "Menor o poca concentración"?

0                                                                                                                               10

¿Qué tan fácil es la comprensión del ítem N° 18 "Frustración, cansancio, irritabilidad"?

0                                                                                                                               10

¿Qué tan fácil es la comprensión del ítem N° 19 "Triste"?

0                                                                                                                               10

¿Qué tan fácil es la comprensión del ítem N° 20 "Sentirse avergonzado"?

0                                                                                                                               10

 ¿Qué tan fácil es la comprensión del ítem N° 21 "Obstrucción nasal"?

0                                                                                                                               10

¿Qué tan fácil es la comprensión del ítem N° 22 "Perdida del sentido del olfato y gusto"?

0                                                                                                                               10

**Por cada pregunta, superior a 7 se acepta pregunta. 4 a 6 se discute. 1 a 3 cambia. Se tomará el valor más bajo para las decisiones.**

**DATOS SOCIODEMOGRÁFICOS Y CLÍNICOS (TOMADOS DE LAS HISTORIAS CLÍNICAS Y/O COMPLETADOS POR OTORRINOLARINGÓLOGO TRATANTE)**

Nombre:

Edad:

Estrato socioeconómico:

Nivel educativo:

Medicamentos usados:

Antecedentes patológicos:

Cirugías de senos paranasales previas SI 𛲡   NO 𛲡

                                                           SI NO

Bloqueo / Obstrucción nasal           𛲡 𛲡

Congestión nasal                            𛲡 𛲡

Descarga nasal (anterior o posterior).𛲡 𛲡

Dolor / presión facial.                      𛲡 𛲡

Hiposmia / anosmia.                       𛲡 𛲡

Signos endoscópicos (pólipos nasales y/o descarga mucopurulenta desde meato medio y/o edema/obstrucción mucosa).

SÍ 𛲡?   NO   𛲡

Cambios tomográficos (cambios mucosos dentro del complejo ostiomeatal y/o SPN)

SÍ 𛲡   NO   𛲡

**PREGUNTA A DISCUTIR POR EXPERTOS.**

Item "Mucosidad nasal continua"

1. ¿Cambiaría usted este ítem de la escala?

- Si
- No

2. En caso de que fuera necesario cambiarlo ¿por opción lo haría?

- Moco constante en la nariz
- Mucosidad continua en la nariz
- Sensación de tener moco en la nariz
- Secreción nasal continua (versión escala Española)

3. Propone otra opción para cambiar este ítem

- Si
- No

4.     Nombre completo

**Encuesta a expertos tras comprensión del cuestionario y corrección o cambio de Item 3.**

**Relevancia, Suficiencia y Exhaustividad del cuestionario SNOT- 22**

Califique de 1 a 3 los ítems y dominios del cuestionario SNOT -22

1. Innecesario
2. Útil, pero prescindible
3. Escencial

| DOMINIO | ÍTEM | RELEVANCIA  Le parece que el ítem de la escala SNOT 22  es relevante e importante a la hora de evaluar los síntomas rinosinusales | EXHAUSTIVIDAD  ¿Le parece que los ítems abarcan de forma integra los síntomas rinosinusales? | SUFICIENCIA  ¿Le parece que las preguntas son suficientes para evaluar los síntomas rinosinusales? |
| --- | --- | --- | --- | --- |
| NARIZ | N°1 “Necesidad de sonarse la nariz" | 1        2         3 | ¿Le parece que los ítems del dominio “Nariz” abarcan de forma íntegra los síntomas nasales de la rinosinusitis?    1         2         3 | ¿Los ítems que conforman el dominio “Nariz”, son suficientes para evaluar los síntomas nasales de la rinosinusitis?    1         2         3 |
|  | N°2 “Estornudo” | 1        2         3 |  |  |
|  | N°3 “Secreción nasal continua” | 1        2         3 |  |  |
|  | N°4 “Tos” | 1        2         3 |  |  |
|  | N°5 “Cae secreción por atrás hacia la garganta” | 1        2         3 |  |  |
|  | N°6 “Secreción nasal espesa” | 1        2         3 |  |  |
|  | N°21 “Obstrucción nasal” | 1        2         3 |  |  |
| OÍDOS/FACIAL | N°7 “Sensación de oído tapado” | 1        2         3 | ¿Le parece que los ítems del dominio “Oídos/facial” abarcan de forma íntegra los síntomas óticos y faciales de la rinosinusitis?    1         2         3 | ¿Los ítems que conforman el dominio “Oídos/facial", son suficientes para evaluar los síntomas óticos y faciales de la rinosinusitis?    1         2         3 |
|  | N°8 “Mareos” | 1        2         3 |  |  |
|  | N°9 “Dolor de oído” | 1        2         3 |  |  |
|  | N°10 “Presión o dolor en la cara” | 1        2         3 |  |  |
| SUEÑO | N° 11 "Dificultad para quedarse dormido(a)" | 1        2         3 | ¿Le parece que los ítems del dominio “Sueño” abarcan de forma íntegra los síntomas que afectan el sueño en la rinosinusitis?    1         2         3 | ¿Los ítems que conforman el dominio sueño, son suficientes para evaluar los síntomas que afectan el sueño en la rinosinusitis?    1         2         3 |
|  | N° 12 "Se despierta durante la noche" | 1        2         3 |  |  |
|  | N° 13 "Sensación de que durmió mal" | 1        2         3 |  |  |
|  | N° 13 "Sensación de que durmió mal" | 1        2         3 |  |  |
|  | N° 14 "Despertar cansado (a)” | 1        2         3 |  |  |
| FUNCIONALIDAD | N° 15 "Fatiga o cansancio" | 1        2         3 | ¿Le parece que los ítems del dominio “Funcionalidad” abarcan de forma íntegra la afectación funcional de la rinosinusitis?    1         2         3 | ¿Los ítems que conforman el dominio Funcionalidad, son suficientes para evaluar la afectación funcional de la rinosinusitis?    1         2         3 |
|  | N° 16 "Productividad o rendimiento disminuido" | 1        2         3 |  |  |
|  | N° 17 "Menor o poca concentración" | 1        2         3 |  |  |
| EMOCIONES | N° 18 "Frustración, cansancio, irritabilidad" | 1         2         3 | ¿Le parece que los ítems del dominio “Emociones” abarcan de forma íntegra la afectación emocional de la rinosinusitis?  1         2         3 | ¿Los ítems que conforman el dominio Emociones, son suficientes para evaluar la afectación emocional de la rinosinusitis?    1         2         3 |
|  | N° 19 "Triste" | 1         2         3 |  |  |
|  | N° 20 "Sentirse avergonzado" | 1         2         3 |  |  |
| SENTIDO DEL OLFATO Y GUSTO | N° 22 "Perdida del sentido del olfato y gusto" | 1         2         3 | ¿Le parece que estos ítems abarcan de forma íntegra la afectación de estos sentidos en la rinosinusitis?    1         2         3 | ¿Estos items, son suficientes para evaluar la afectación de estos sentidos en la rinosinusitis?  1         2         3 |

Dr (a):_______________________________________

**ESCALA VISUAL ANÁLOGA. SÍNTOMAS NASOSINUSALES**

Gravedad de los síntomas: Por favor dibuje una línea vertical en el punto que mejor corresponda, según que tan molestos sean los siguientes síntomas en los últimos meses. Según el ejemplo.

**
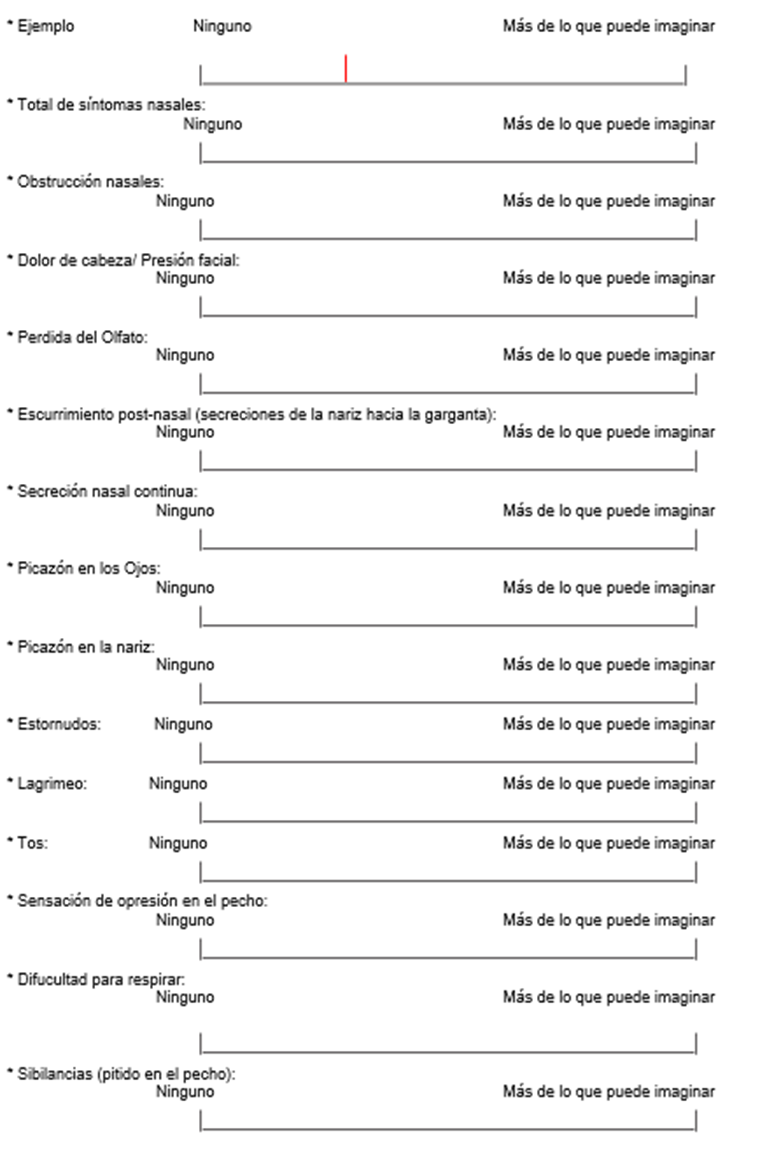
**

**MATERIAL COMPLEMENTARIO. RESULTADOS**

**CUESTIONARIO SNOT-22 AJUSTADO.**

Fecha:

A continuación, usted encontrará una serie de síntomas y alteraciones socio/emocionales asociadas a su rinosinusitis. Nos gustaría saber más acerca de estos problemas y le solicitamos contestar las siguientes preguntas, lo mejor posible. No hay respuestas correctas o incorrectas y sólo usted nos puede entregar esa información. Por favor, califique sus molestias según cómo han sido estas últimas dos semanas. Gracias por su participación. No dude en pedir ayuda sí la necesita.


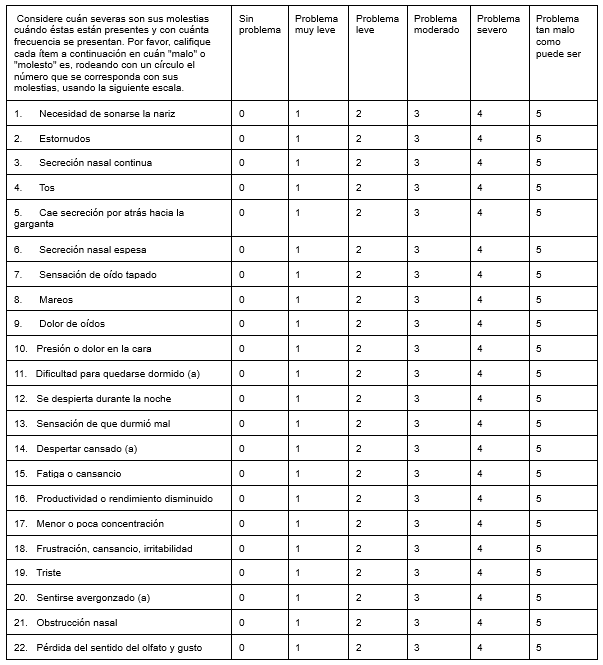


**Puntaje total___________________________________________________**

**RESULTADOS. Material Suplementario.**

| **Tabla 1. Material Suplementario.**  **Comprensión por Expertos.** | | | | | | |
| --- | --- | --- | --- | --- | --- | --- |
| **DOMINIO** |  | **ÍTEM** | **ENUNCIADO** | **PROMEDIO COMPRENSIÓN ADAPTADO** | **PROMEDIO EN PRUEBA PILOTO** | **PUNTAJE MÁS BAJO** |
| ENCABEZADO |  |  |  | 8.267 | 7.4 | 3 |
| NARIZ |  | Ítem 1 | “Necesidad de sonarse la nariz" | 10 | 10 | 10 |
|  |  | Ítem 2 | “Estornudo” | 10 | 10 | 10 |
|  |  | Ítem 3 | “Secreción nasal continua” (en piloto, mucosidad nasal continua” | 9.5 | 6.4 | 7 (2) |
|  |  | Ítem 4 | “Tos” | 9.87 | 9.6 | 8 |
|  |  | Ítem 5 | “Cae secreción por atrás hacia la garganta” | 8.8 | 8.2 | 4 |
|  |  | Ítem 6 | “Secreción nasal espesa” | 9.47 | 9.4 | 7 |
|  |  | Ítem 21 | “Obstrucción nasal” | 9.87 | 10 | 8 |
| OÍDOS/FACIAL |  | Ítem 7 | “Sensación de oído tapado” | 10 | 10 | 10 |
|  |  | Ítem 8 | “Mareos” | 9.33 | 9.6 | 5 |
|  |  | Ítem 9 | “Dolor de oído” | 10 | 10 | 10 |
|  |  | Ítem 10 | “Presión o dolor en la cara” | 9.87 | 9.8 | 9 |
| SUEÑO |  | Ítem 11 | "Dificultad para quedarse dormido (a)" | 9.67 | 9.2 | 8 |
|  |  | Ítem 12 | "Se despierta durante la noche" | 9.53 | 8.8 | 6 |
|  |  | Ítem 13 | "Sensación de que durmió mal" | 9 | 8.2 | 3 |
|  |  | Ítem 14 | "Despertar cansado (a)” | 9.2 | 8.2 | 2 |
| FUNCIONALIDAD |  | Ítem 15 | "Fatiga o cansancio" | 9.4 | 9 | 7 |
|  |  | Ítem 16 | "Productividad o rendimiento disminuido" | 9.13 | 8.6 | 6 |
|  |  | Ítem 17 | "Menor o poca concentración" | 9.4 | 9 | 7 |
| EMOCIONES |  | Ítem 18 | "Frustración, cansancio,  irritabilidad" | 9.53 | 9.2 | 8 |
|  |  | Ítem 19 | "Triste" | 9.27 | 7.8 | 2 |
|  |  | Ítem 20 | "Sentirse avergonzado" | 9.27 | 8.2 | 4 |
| SENTIDO DEL OLFATO Y DEL GUSTO |  | Ítem 22 |  |  |  |  |

| **Tabla 2 Material Suplementario. Índice de Validez de Contenido SNOT-22 adaptado** | | |
| --- | --- | --- |
| IVC de Relevancia | IVC de Suficiencia | IVC de Exhaustividad |
| 0.658 | 0.744 | 0.698 |

*IVC: Indice de Validez de Contenido*

| **Tabla 3. Material Suplementario. Índices de Bondad de Ajuste** | | | | |
| --- | --- | --- | --- | --- |
| RMSEA | Root Mean Square Error of Approximation | | | 0.081 (IC 0.071-0.090) |
| CFI | Comparative Fit Index | |  | 0.979 |
| TLI | Tucker-Lewis Index | |  | 0.975 |
| SRMR | Standardized Root Mean Square Residual | | | 0.049 |
| X^2^ | Chi Cuadrado |  |  | 483.116 |
|  | Valor de P |  |  | 0.000 |
|  | Grados de libertad | |  | 199 |


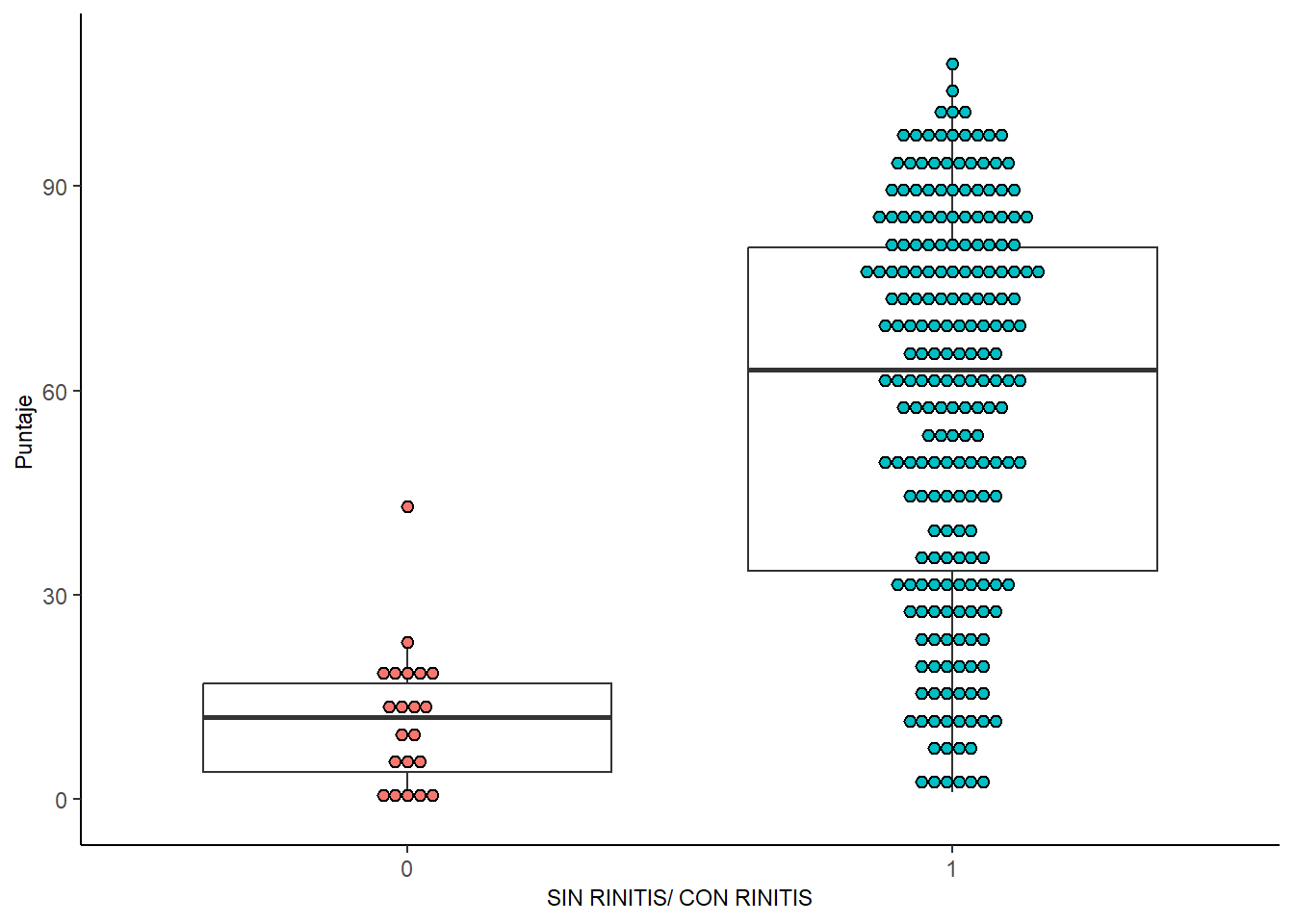


Without CRS

With CRS

**Mean Score SNOT-22**

People without CRS: 11.66

People with CRS: 57.90

**Figura.** Dot plot de puntajes de SNOT-22 en pacientes con rinosinusitis y pacientes sin rinosinusitis
